# The relationship between headache-attributed disability and lost productivity: 1. A review of the literature

**DOI:** 10.1186/s10194-021-01264-0

**Published:** 2021-07-17

**Authors:** Simple Futarmal Kothari, Rigmor Hølland Jensen, Timothy J Steiner

**Affiliations:** 1 Section of Orofacial Pain and Jaw Function, Department of Dentistry and Oral Health, Aarhus University, Aarhus, Denmark; 2 Hammel Neurorehabilitation Centre and University Research Clinic, Hammel, Denmark; 3 Danish Headache Centre, Department of Neurology, University of Copenhagen, Rigshospitalet Glostrup, Glostrup, Denmark; 4 Department of Neuromedicine and Movement Science, NTNU Norwegian University of Science and Technology, Edvard Griefs gate, Trondheim, Norway; 5 Division of Brain Sciences, Imperial College London, London, UK

**Keywords:** Headache disorders, Disability, Lost productivity, Systematic literature review, Global Campaign against Headache

## Abstract

**Background:**

Headache disorders are disabling and have a significant impact on productivity. The relationship between these two consequences is of considerable economic and political interest. We enquired into it through a systematic search of the English-language literature.

**Methods:**

We followed PRISMA guidelines in specifying search terms and syntax and in article selection. We used the term “disability” in the search, accepting any meaning that authors attached to it, but this proved problematic. Accordingly, we adopted the definition used in the Global Burden of Disease study. In article selection, we included only those that purported to measure disability as so defined and lost productivity. We reviewed the full texts of those selected. We included further articles identified from review of the bibliographies of selected articles.

**Results:**

The literature search found 598 studies, of which 21 warranted further review. Their bibliographies identified another four of possible relevance. On full-text reading of these 25, all were rejected. Ten applied incompatible definitions of disability and/or lost productivity. Two did not measure both. Four reported lost productivity but not disability. Eight studies reported and measured both but did not assess the association between them or provide the means of doing so. One was purely methodological.

**Conclusions:**

The literature is silent on the relationship between headache-attributed disability and lost productivity. In view of its health economic and political importance, empirical studies are required to remedy this. A prerequisite is to clarify what is meant by “disability” in this context.

## Background

There is no doubt that headache disorders are disabling [1–4]. Disability is the consequence of the symptoms of headache disorders, including pain of course but also others such as nausea and photophobia, which, separately or together, are debilitating and sometimes prostrating [5].

The Global Burden of Disease study (GBD) in its multiple iterations shows that three headache disorders – migraine, tension-type headache (TTH) and medication-overuse headache (MOH), which is a sequela of migraine or TTH – are major contributors to public ill health [2–4]. GBD2016 (not the most recent iteration, but the one with the most detailed analyses of headache [3]) reported almost 3 billion people affected by headache disorders: over 1.9 billion with TTH and another 1 billion with migraine. Global age-standardized prevalences were 26.1 % for TTH and 14.4 % for migraine, although these varied considerably through the different world regions. In GBD2019, the most recent to be analysed, headache disorders were estimated to be responsible for 46.6 million years lived with disability (YLDs) globally (5.4 % of all YLDs), with an age-standardized rate of 602.5 YLDs/100,000 person-years [4, 6]. These estimates ranked headache disorders as the third cause of disability (after back pain and depressive disorders), and first cause in adults under 50 years [4, 6].

What does this actually mean? GBD has recently been described as “the largest and most comprehensive effort to quantify health loss across places and over time” [7]. Note the term *health loss*. GBD uses two principal metrics: YLDs and years of life lost (YLLs) through premature mortality, which are summed as disability-adjusted life years (DALYs) [8]. We say no more about YLLs since these have no relevance to headache disorders. YLDs are applied to each of the health states that arise as a consequence of living with a particular disorder, and calculated at individual level according to the proportion of time spent in each health state and the disability weights (DWs) attributed to these. DWs are estimated through a global consultation exercise in which respondents receive lay descriptions of two hypothetical people with randomly selected health states and are asked which of the two is healthier. There is then a complex ranking exercise ordering all health states. A separate grounding exercise, also involving a global consultation, ties the ranking to a scale 0–1, where 0 represents no loss of health and 1 a loss of health equivalent to being dead. YLDs for each health state of a particular disorder are summed. At population level, YLDs are the product of prevalence of the disorder in the population and the mean individual estimate [8].

For episodic headache disorders such as migraine and TTH, two essential health states are recognised: ictal (during attacks) and interictal (between attacks) [5, 9]. Despite clear evidence of lost health during the interictal state, especially of migraine [10], current GBD methodology has not been able to take account of it: while DWs are not sufficiently sensitive to reflect very low levels of lost health, the very large proportion of time spent in the interictal state (typically > 90 %) greatly magnifies the inaccuracy. So, for migraine for example, population-level YLDs are the product of its prevalence, mean time spent in the ictal state (itself a product of the means of attack frequency and duration) and a DW of 0.441 [9, 11]. For TTH, the DW is a much lower 0.037 [11]. To the extent that MOH is a sequela of migraine or TTH, it is a third health state of each, and GBD assigns YLDs attributable to MOH (with ictal DW = 0.217 [11]) proportionately to migraine and TTH [3].

This is how the numbers above were derived, but they are not estimates of *disability* as this term is generally understood (impact of impairment on a person’s functional ability [12, 13]). This point was highlighted a decade ago by Grosse et al. [14], and by Mathers et al. several years earlier: “The disability weights used in DALY calculations quantify *societal preferences for different health states* [emphasis added] … in relation to the societal ‘ideal’ of optimal health” [15]. As Mathers et al. went on to explain, “on average, society judges a year with blindness (weight 0.43) to be preferable to a year with paraplegia (weight 0.57), and a year with paraplegia to be preferable to a year with unremitting unipolar major depression (weight 0.76)” [15]. So YLDs – if sound in their construct validity (which is essentially dependent on the correct valuation of DWs [11]) – are an expression of ill health in a much more comprehensive sense than is conveyed by the term *disability*.

Many studies estimating the burdens attributable to headache disorders have used the Migraine Disability Assessment (MIDAS) questionnaire [16] or its derivatives, the Headache-Attributed Lost Time (HALT) indices [17]. Note the different terminology in the names of these essentially similar measures. They use the same questions, although worded slightly differently, to estimate days lost from work, through presenteeism as well as absenteeism, and from household chores, and lost or curtailed social occasions. These losses may be consequences of *ill health* but they are, again, not *disability* as generally understood [12, 13]. They are subject to *choice*: choice, unless it is overcome by total prostration, plays a large part in determining what is done or not done in the face of impairments such as pain. Choice is itself influenced by a multiplicity of factors. More will be said later about choice.

The ill health associated with headache disorders, and the disability that is its consequence, inevitably lead to lost productivity [5]. There is workforce-based evidence of this. For example, 968 (16.4 %) of 5,940 employees engaged in car manufacture in Turkey reported productivity losses attributed to headache totalling 6,452 h/week (increasing with headache frequency from 0.23 to 7.6 days/month per affected employee) [18]. More broadly, population-based evidence attests to national productivity losses: in China, 1.3 % of gross domestic product (GDP) is reportedly lost to headache [19], in Ethiopia 1.6 % [20], in Zambia 1.9 % [21], in India 3 % [22] and in Nepal 5.6 % [23]. Relevant here is that migraine most affects those aged 15–49 years, the productive years, when families and careers are built, and prospects established for the rest of life [24]. As already noted, headache disorders are the top cause of YLDs in this age-group [4, 6]. Countries with younger populations are likely to be affected disproportionately highly.

The importance of all this is obvious as a major public-health concern, but there is another crucial consideration. Lost productivity represents economic loss (so-called indirect cost), and lost productivity on the scale reflected in these countries’ GDPs represents very substantial economic loss. The Eurolight project, a survey conducted in ten European countries, estimated societal losses attributable to all headache disorders (direct and indirect costs) at well over €100 billion per year, with more than 90 % attributable to lost productivity [25]. The World Health Organization (WHO) observed in 2011 that, if only part of this lost productivity could be recovered through better treatment, investments in improved health services delivering better headache care would not only be highly cost-effective but might be cost saving [26].

There were three assumptions in that assertion. The first, that headache care alleviates the ill health associated with headache disorders, and the second, that it does this more effectively if delivered more widely by “better” health services [24], can reasonably be accepted as truisms. The third, however, that this will lead to a commensurate recovery of headache-attributed lost productivity, is far from certain. It is intuitively true, but the large element of *choice* referred to earlier in what is done or not done when a person is affected by headache casts doubt upon it. It requires support from empirical evidence, and our purpose in this review of the literature is to discover whether this evidence exists.

## Methods

We conducted a systematic review of the English-language literature evaluating the relationship between headache-attributed ill health (expressed as symptom burden or disability) and lost productivity. We followed PRISMA (Preferred Reporting Items for Systematic Reviews and Meta-Analyses) guidelines in specifying search terms and syntax and in article selection.

### Search strategy and selection criteria

We performed an online search in August-September 2017 of the PubMed database, with no start date, using Medical Subject Headings (MeSH) terms “headache” or “headache disorders” and “cost of illness” or “absenteeism” or “presenteeism” or “sick leave”. “Disability” did not exist as a MeSH term, so a free text-based search was also included. The full statement (terms and syntax) is set out in Table 1.

**Table 1:** Search terms and syntax

{("Headache"[Mesh] OR "Headache Disorders"[Mesh] OR headache*[Text Word] OR migraine*[Text Word]) AND ("Cost of Illness"[Mesh] OR "Absenteeism"[Mesh] OR "Presenteeism"[Mesh] OR "Sick Leave"[Mesh] OR productivity[Text Word] OR productive time[Text Word] OR absenteeism[Text Word] OR presenteeism[Text Word] OR sick leave[Text Word] OR indirect cost[Text Word] OR MIDAS[Text Word]) AND (disabilit*[Text Word] OR frequency[Text Word] OR duration[Text Word]) AND English[lang]} {NOT ("Animals"[Mesh] NOT "Humans"[Mesh])} {NOT ("Review" [Publication Type] OR "Meta-Analysis" [Publication Type] OR "Letter" [Publication Type] OR "Congresses" [Publication Type] OR "Case Reports" [Publication Type])}	

#### Inclusion criteria

In the initial search, *lost productivity* was defined to include days of absenteeism from or < 50 % productivity in paid or household work (the MIDAS construct [16]), estimated either over the preceding 3 months or in association with headache yesterday [5]. We took the term *disability* to have any meaning attached to it in the literature, but in the subsequent selection of articles this was problematic. Much of the literature used the term without explicit meaning, but two principal definitions emerged: those attributed by MIDAS [16] and GBD [8, 9, 11, 14, 15]. The former predominated, but its definition of “disability” was exactly as we defined “lost productivity”, and therefore unusable. The search encountered several additional uses of the term. WHO’s International Classification of Functioning, Disability and Health (ICF) [27–29] is a framework using disease-specific question sets to evaluate an individual’s ability to participate in activities within his or her specific environmental constraints [27, 28]. While this might seem a comprehensive account of disability, it is descriptive rather than quantitative, and therefore not amenable to statistical association analysis. In any case, no ICF headache question set existed [29]. The HEADWORK questionnaire assesses disability, but narrowly, with respect to a limited number of specific work-related tasks [30, 31], several not relevant to large labour sectors (such as manual work and farming – especially important in low-income countries). The Headache Impact Test (HIT-6) also appeared, but its six questions (four of which arguably relate to disability) do not provide quantitative estimates [32]. This left only the GBD definition. In article selection, therefore, we defined *disability* restrictively, only in this sense, notwithstanding our reservations about it expressed earlier. This definition did have two major advantages: it lends itself readily to economic analysis, and carries the *imprimaturs* of the World Bank, WHO and the Institute for Health Metrics and Evaluation (IHME), who conduct GBD [33].

We included studies that purported to measure disability so defined *and* lost productivity defined only as above. Studies that measured duration and frequency of headache episodes along with lost productivity were also included since YLDs were calculable.

### Review

From our initial search, articles labelled as reviews, case reports, animal studies, lectures, guidelines or randomized controlled trials were excluded. Those remaining were imported into a reference management software (Zotero, Center for History and New Media, George Mason University, Fairfax, VA, USA) [34] and duplicates were removed.

In further screening, studies were sorted on the basis of their titles and abstracts. Those that appeared potentially relevant were retrieved as full papers, as were those that might, from their titles, meet our eligibility criteria but were insufficiently informative in their abstracts. The search was extended to the bibliographies of articles obtained in full text.

All full-text articles found in this process were scrutinised for evidence or comment regarding the relationship between disability and lost productivity.

## Results

**Fig. 1:**
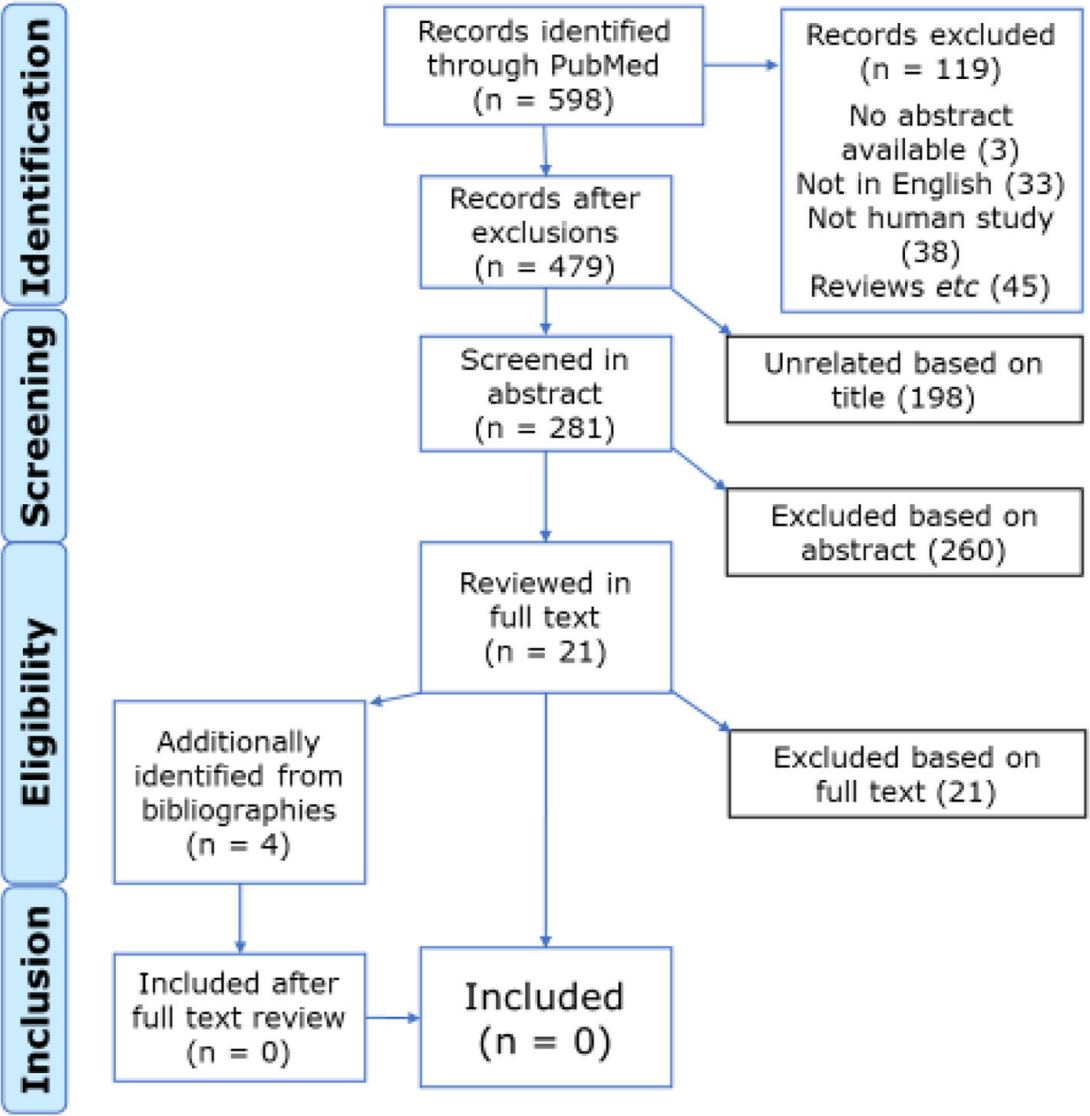
PRISMA flow diagram of article selection

Figure 1 shows the flowchart for article selection according to PRISMA guidelines [35, 36]. The search generated a total of 598 titles. The full texts of 21 papers were obtained for further review. The bibliographies of these suggested four more studies according to their titles, and full texts of these were also obtained after review of their abstracts.

Of the 25 articles selected, all were subsequently rejected. Ten applied different definitions of headache-attributed disability and/or lost productivity, incompatible with ours [37–46]. Two did not measure both [47, 48]. Four measured lost productivity according to our definition, but did not report disability [10, 49–51]. Eight studies reported and measured both as we defined them, but did not assess association between the two or provide the means of doing so [19–23, 52–54]. One was a purely methodological report [5].

In other words, the literature was entirely silent with regard to the relationship between headache-attributed disability and lost productivity.

## Discussion

Our systematic review of the literature, with the enquiry framed broadly, found not just that it said nothing on the relationship between headache-attributed disability and lost productivity but that this issue of substantial health-economic importance had not even been addressed [9, 24–26]. There were confusing and conflicting definitions of “disability” that harnessed this term to both sides of the relationship.

The implications for economic analyses and informed health policy with regard to headache services are considerable. Elsewhere there is an abundant literature on the ill-health burden of headache disorders (e.g., [1–6, 10, 18–23, 47–54]). There is a wealth of empirical evidence of the lost productivity burden of headache and its economic consequences (e.g., [18–25]), to which indirect costs (essentially from lost productivity) are far more contributory than direct health-care costs [25]. There is a huge volume of evidence, too large to recount, that migraine, TTH and MOH can be treated effectively. Economic analyses find many treatments for headache to be, at least potentially, highly cost-effective [55, 56]. But all these are not enough [6, 24, 57]. What is needed to empower the economic argument for prioritising headache care within health services [6, 24, 26] and transform it into a persuasive political argument is evidence that effective treatment of headache does actually recover at least part of the headache-attributed lost productivity [24, 26, 56]. An attempt to demonstrate this empirically in a heavy construction workforce in the Turkish motor industry, where production losses from headache were very high [18], was unsuccessful not because headache care failed to achieve this but because the unconvinced workforce failed to take up the offer of free on-site care [50]. That the literature offers no help either is disappointing.

It is also a prompt for studies to remedy the deficit. When these are attempted, clarity in definitions is a prerequisite. The GBD studies measure lost health due to headache as proportion of time in the ictal state (pTIS, calculated as attack frequency * mean duration) [2–4, 6] and express the product of pTIS and the DW for the ictal state [11] in YLDs – hence “disability”, but not, as noted, in the usual sense of this term [14, 15]. For both migraine and TTH, with lost-health estimates based on a single (ictal) health state, DW is a constant: eliminating it will not change relationships. If pain intensity were introduced in its place (pTIS * mean intensity) to generate what might be considered a measure of symptom burden – *impairment* rather than “disability” – the result would arguably be a more nuanced measure of health loss, which might relate to lost productivity more closely and offer a better way forward in future studies. Pain intensity is highly subjective, but function may be sensitively attuned to it for this very reason. For the same reason, however, it is not easily or reliably quantified [5].

Lost productivity is readily defined conceptually. As a construct, however, it is complex. It is complicated by its separable elements: losses from earnings-generating work, from necessary household and other life- and lifestyle-maintaining chores, and from social life [5, 16, 17]. It is complicated further by its influencers. Best described as a behavioural response to impairment [5], lost productivity is a consequence of *choice* – a subject we indicated earlier that we would return to. Whether to commence, continue or abandon work (of whatever nature) when impaired by headache is a choice, unless impairment is total [5]. Factors on which this choice depends may be disease-related: the severity of impairment, obviously, but responses might not be the same to occasional and unexpected attacks as to more predictable frequent episodes. Other factors, however, have more to do with culture, with personal characteristics such as lifestyle, stoicism and general health, or with socioeconomic conditions such as employment level, potential loss of pay and fear of job loss. Some are directly work-related – to its nature, necessity and enjoyability. Some, such as intemperate weather, are entirely random. Reduced productivity while engaged in work with headache (“presenteeism”, in the context of paid employment) commonly accounts for more lost productivity than absenteeism [18, 38, 39, 45, 58], with, possibly, different determinants. All of these intrude into and are likely to camouflage any relationship that might exist between disability and lost productivity.

Some population studies have found that lost productivity due to headache (in percentage time units) exceeded percentage disability estimated as pTIS * DW [20, 53]. This might indicate that DWs are too low, especially the 0.037 for TTH [11, 20], or that the disabling effect of headache outlasts it. Significant interictal burden is reported by many people with migraine, and some with TTH [5, 10]. An Indian study described motivation and energy lost during migraine, symptoms that might for some time outlast the ictal state [53]. Factors such as these are further complications in any relationship.

This study had no important limitations. The search was restricted to English-language publications, and to PubMed, in which we expected to find anything noteworthy and of relevance. It was otherwise comprehensive, with inclusive search terms. The problems that required us to define “disability” restrictively were limitations of the data, not of the study methodology.

## Conclusions

A careful search of the English-language literature found nothing on the relationship between headache-attributed disability and lost productivity. In view of the substantial health economic and therefore political importance of this, it is a deficit that needs remedy in empirical studies. A prerequisite is to clarify what is meant by “disability”.

## Data Availability

Not applicable.

## Abbreviations

DALY: Disability-adjusted life year
DW: Disability weight
GBD: Global Burden of Disease (study)
GDP: Gross domestic product
HALT: Headache-attributed lost time (indices)
MeSH: Medical Subject Headings
MIDAS: Migraine disability assessment (questionnaire)
MOH: Medication-overuse headache
PRISMA: Preferred Reporting Items for Systematic Reviews and Meta-Analyses
pTIS: Proportion of time in ictal state
TTH: Tension-type headache
WHO: World Health Organization
YLD: Year lived with disability
YLL: Year of life lost
